# Karyotype and chromosomal characteristics of rDNA of *Cobitisstrumicae* Karaman, 1955 (Teleostei, Cobitidae) from Lake Volvi, Greece

**DOI:** 10.3897/CompCytogen.v12i4.28068

**Published:** 2018-11-16

**Authors:** Eva Hnátková, Costas Triantaphyllidis, Catherine Ozouf-Costaz, Lukáš Choleva, Zuzana Majtánová, Joerg Bohlen, Petr Ráb

**Affiliations:** 1 Department of Zoology and Fisheries, Faculty of Agrobiology, Food and Natural Resources, Czech University of Life Sciences, 165 00 Prague, Kamýcká 129, Czech Republic Czech University of Life Sciences Prague Czech Republic; 2 Department of Genetics, Development and Molecular Biology, Faculty of Sciences, School of Biology, Aristotle University of Thessaloniki, University Campus, 54124 Thessaloniki, Greece Aristotle University of Thessaloniki Thessaloniki Greece; 3 Institut de Biologie Paris Seine, UMR 7138 “Evolution”, Sorbonne Universités, Case 5, 7 quai St Bernard, 75952 Paris cedex 05, Paris, France Sorbonne Universités Paris France; 4 Laboratory of Fish Genetics, Institute of Animal Physiology and Genetics, Academy of Sciences of Czech Republic, 277 21 Liběchov, Czech Republic Institute of Animal Physiology and Genetics, Academy of Sciences of Czech Republic Libĕchov Czech Republic

**Keywords:** chromosome banding, NOR phenotype, FISH, rDNA, cytotaxonomy of *Cobitis* loaches

## Abstract

The karyotype of Greek cobitid fish *Cobitisstrumicae* Karaman, 1955, from Lake Volvi, Greece, a representative of one of its two major intraspecific phylogenetic lineages, was analysed by means of sequential Giemsa-staining, C-banding, silver-staining, CMA_3_ fluorescence banding and also by *in situ* hybridization (FISH) with rDNA probe. The diploid chromosome number was 2n = 50, karyotype composed of 10 pairs of metacentric to submetacentric and 15 pairs of subtelocentric to acrocentric chromosomes. The nucleolus organizer regions (NORs) as revealed by Ag- and CMA_3_ staining and FISH were situated in the telomeric region of the fourth submetacentric chromosome pair. The chromosomes contained very low content of C-positive heterochromatin. No heteromorphic sex chromosomes were detected. This first karyotype report for any species of lineage *Bicanestrinia* Băcescu, 1962 shows a simple karyotype dominated by acrocentric chromosomes and possessing single NOR-bearing chromosome pair. Cytotaxonomic implications of this finding for the taxonomy of the genus *Cobitis* Linnaeus, 1758 are further discussed.

## Introduction

The genus *Cobitis* Linnaeus, 1758 attracted the interest of evolutionary biologists by producing several gynogenetic female-only lineages after hybridisation of species (Bohlen and Ráb 2001). As reasons for the asexual reproduction in these hybrids differences in the karyotype and chromosome structure between the parental species have been proposed. Indeed, within *Cobitis* a large variability of karyotypes and chromosomal markers have been observed (Janko et al. 2007). On the other hand, species of *Cobitis* are morphologically highly similar and difficult to identify on the basis of morphologic characters. They have a pronounced sexual dimorphism with males being smaller than females and developing an ossified plate-like structure on the dorsal side of the pectoral fins, called ‘*lamina circularis*’. The widespread presence of hybrid lineages further complicates the systematics and taxonomy of *Cobitis* loaches, therefore genetic methods are applied in identification of species. Chromosome studies have shown that most species have a diploid chromosome number of 2n = 50, but highly diversified karyotypes (reviewed in Ráb and Slavík 1996, Arai 2011). This genetic marker therefore appears to be one of the key parameter in the genetic and taxonomic studies of *Cobitis* loaches, e.g. Ráb and Slavík (1996), Boroň and Danilkiewicz (1998), Vasil’eva and Vasil’ev (1998), Ráb et al. (2007), and serves as one of the determination tools to identify genome composition in hybridogenous clonal asexual biotypes (Janko et al. 2007, Majtánová et al. 2016).

Recent phylogenetic studies (Buj et al. 2014, Ludwig et al. 2001, Perdices and Doadrio 2001, Perdices et al. 2016) demonstrated that the European representatives of *Cobitis* include five major lineages, namely the ‘Siberian lineage’, represented by a single species *C.melanoleuca* Nichols, 1925, Băcescu's (1962) subgenera *Acanestrinia* (now often referred to as ‘Adriatic lineage’), *Iberocobitis*, *Bicanestrinia*, and Cobitis s. str. The subgenus Bicanestrinia is morphologically well characterized by having two *laminae circulares* on the pectoral fins of males. Species of *Bicanestrinia* occur in the Middle East (Turkey, Iran, Syria) and southeast Europe (Bulgaria, Greece) (Bohlen et al. 2006). Up to now, only one species of *Bicanestrinia*, *C.linea* (Heckel, 1847), has ever been analysed in a cytogenetic study, therefore little is known about cytogenetic similarities and differences between *Bicanestrinia* and *Cobitis* s. str. One of the European species of *Bicanestrinia*, *C.strumicae* Karaman, 1955, has long been known from rivers draining into the Aegean Sea, such as Struma, Maritza and the lakes adjacent to the Struma basin such as Volvi and Koronia in Greece. However, it has recently been found in the Danube basin, where it is genetically involved in asexual hybrid forms (Choleva et al. 2008). Since further studies on this example of a sperm-dependent hybrid switch of the sexual hosts require a proper identification of the genetic material of *C.strumicae*, the cytogenetic analysis of Struma spiny loach will complete identification tool box of hybrid biotypes of the genus *Cobitis*.

This study reports on the karyotype and other chromosomal characteristics of Greek cobitid fish *C.strumicae* from population inhabiting Lake Volvi, Greece, analysed by means of sequential Giemsa-staining, C-banding, silver-staining, CMA_3_ fluorescence banding and by *in situ* hybridization (FISH) with 28S rDNA.

## Material and methods

Ten males and two females were collected at the outlet of a thermal spring into Lake Volvi, Greece, by dip net and transferred alive to the laboratory. The examined specimens are deposited as voucher samples in the collection of the Laboratory of Fish Genetics, IAPG, CAS, Liběchov, under Accession Code CoS/97. Valid Animal Use Protocol was in force during study in IAPG (No. CZ 02386). Standard procedures for chromosome preparation followed Ráb and Roth (1988). Silver (Ag-) staining and Chromomycin A_3_ (CMA_3_) fluorescence banding, for detection of NORs, followed Howell and Black (1980) and Sola et al. (1992), respectively. The sequence of stainings followed protocol of Rábová et al. (2016). Fluorescence *in situ* hybridization (FISH) with a mouse rDNA biotinylated probe (clone I-19, a 4.2-kb EcoRI-SalI fragment containing most of the 28S rDNA-coding region) to detect chromosomal sites of rDNA, i.e. sites of NORs, followed the procedure of Reed and Phillips (1995) and Ozouf-Costaz et al. (1996). Briefly, a mouse rDNA clone, was biotin-labelled by nick translation (Oncor, Inc). Chromosomes were pretreated by incubating the slides in 2X SSC (pH 7.0) at 37 °C for 30 min, dehydrated in a 4 °C ethanol series, and air-dried. Chromosomal DNA was denatured by incubating the slides in a filtered 70% formamide/2X SSC solution (pH 7.0) at 70 °C for 2 min, followed by dehydration in 4 °C ethanol series. Labelled probe was diluted to 16.6 ng/µl in hybridization solution (Hybrisol VII, Oncor; 50% formamide), denatured by incubation at 70 °C for 5 min and placed immediately on ice until applied to slides. Hybridization was performed using 20–25 µl (~ 250 ng) of probe mixture/slide and incubated overnight in a 37 °C humidity chamber. After hybridization, slides were washed in a 50% formamide/2X SSC solution (pH 7.0) at 37 °C for 15 min, followed by an 8 min wash in 2X SSC (37 °C, pH 7.0). Slides were washed at room temperature for 2 min each in the following series: 4X SSC; 4X SSC + triton X; and a 1:1 mix of 4X SSC and PN buffer (0.1 M NaHP0, 0.1 M NaH_2_P0, 5% NP-40 detergent, pH 8.0). Fluorescein-isothiocyanate (FITC)-conjugated avidin was used to detect hybridization signal. Chromosomes were counterstained with propidium iodide (0.375 µg/ml) in antifade (10 mg/ml p-phenylenediamine dihydrochloride (DAPI) in PBS/90% glycerol, pH 8.0) and chromosomes viewed under epifluorescence.

**Figure 1.**
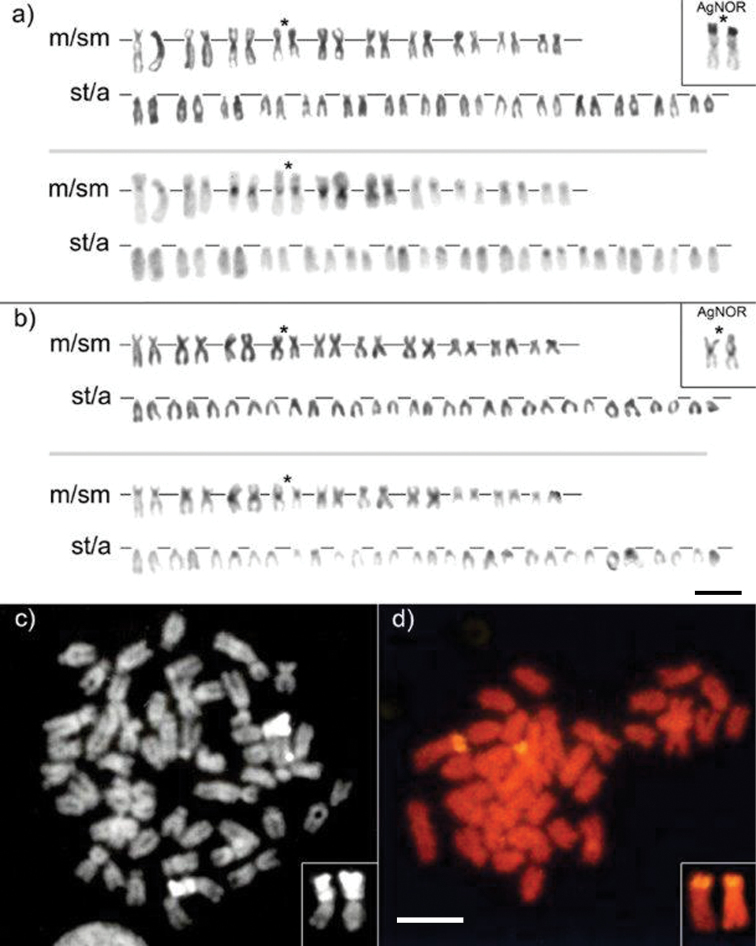
Karyotypes of a male (**a**) and female (**b**) of *C.strumicae* from Lake Volvi arranged from sequentially Giemsa-stained (upper row) and C-banded (lower row) chromosomes; sequentially Ag-stained chromosome pair with positive signal is framed in (**a**) and (**b**); metaphase cells of *C.strumicae* after CMA_3_ staining (**c**) and FISH with rDNA probe (**d**) chromosomes bearing CMA_3_ and FISH signals are framed; m – metacentric, sm – submetacentric, st – subtelocentric and a – acrocentric chromosomes. Scale bar: 10 μm.

At least 25 Giemsa-stained or banded metaphases plates per individual were examined, most of them sequentially. Chromosomes were classified according to Levan et al. (1964), metacentric to submetacentric and subtelocentric to acrocentric chromosomes, respectively were grouped together in Fig. 1.

## Results

### Karyotype and banding analysis

Chromosome counts from all 12 individuals revealed an invariable diploid chromosome number 2n = 50. The karyotype consisted of 10 pairs of metacentric (m) to submetacentric (sm) and 15 pairs of subtelocentric (st) to acrocentric (a) chromosomes (Fig. 1a, b). No heteromorphic sex chromosomes were detected in males (Fig. 1a) and females (Fig. 1b). The nucleolus organizer regions (NORs) as revealed by Ag- and CMA_3_ staining were situated in the telomeric region of the fourth m/sm chromosome pair. This pair of chromosomes was also observed to be end-to-end associated in some metaphases. No variation in number of NORs was observed while size polymorphism was frequently detected. C-banding revealed small heterochromatic blocks in pericentromeric regions of all pairs of chromosomes except fifth and sixth m pairs where the blocks of heterochromatin were large (Fig. 1).

### Chromosomal location of rDNA

FISH with 28S rDNA probe showed strong labelling of a single chromosomal pair (Fig. 1d). Identification of chromosomes by propidium iodide counterstaining revealed the labelled pair to be the same as that identified by Ag- and CMA_3_ staining. No other positively labelled chromosomal sites were found.

### Chromosomal organization of NOR sites

CMA_3_ staining revealed the positive signal on the NOR-bearing pair only (Fig. 1c). The CMA_3_ positive blocks covered entire p arm from the pericentromeric region to telomeres with distinct gap close to centromere. However, C-banding showed positive heterochromatin blocks in pericentromeric region which clearly corresponded to smaller CMA_3_-positive blocks (Fig. 1a, b). Ag-staining (Fig. 1a, b) and FISH (Fig. 1d) showed positive signals in distal parts of shorter arm only.

## Discussion

### Arrangement of nucleolar ribosomal DNA in *C.strumicae* chromosomes

We examined chromosomes of *C.strumicae* by means of several banding methods detecting sites of major ribosomal DNA, i.e. sites of NORs (Ráb et al. 1996). The application of GC-specific fluorochromes such as CMA_3_ or Mithramycin (MM), together with enhancing AT-specific counterstains that specifically interact with GC-rich DNA sequences and/or examination of rDNA loci by FISH indicate that the sites of NORs of teleostean fishes detected by means of silver staining contain large fractions of GC-rich DNA, e.g. Mayr et al. (1985), Amemiya and Gold (1986), Schmid and Guttenbach (1988) and reviewed by Gornung (2013). The association of GC-rich DNA type of heterochromatin with rDNA sites is present in lower and higher teleostean groups, suggesting that it is evolutionarily conserved among teleosts (Gornung 2013). However, this character exits also in bichirs (Polypteriformes), partly in paddlefishes (Symonová et al. 2017a), gars (Symonová et al. 2017b) and bowfin (Majtánová et al. 2017), but not in sturgeons (Fontana et al. 2007). Among *Cobitis* loaches, this characteristic pattern was found in *C.vardarensis* Karaman, 1928 (Rábová et al. 2001), *C.elongatoides* Băcescu et Mayer, 1969 (Ráb et al. 2000) and *C.taenia* Linnaeus, 1758 (Boroň et al. 2006), i.e. species from *Cobitis* s. s. clade. Our analysis of chromosomal characteristics of major rDNA in *C.strumicae* confirms such characteristic association of GC-rich DNA and sites of NORs for the so far uninvestigated subgenus Bicanestrinia.

Recent cytogenetic studies in fish (Gornung 2013), also suggested that not all CMA_3_-positive signals represent sites of NORs but exclusively GC-rich heterochromatin blocks which are not associated with ribosomal DNA (Ráb et al. 1996). Our investigation of *C.strumicae* chromosomes using several methods to detect NORs revealed such type GC-rich DNA heterochromatin which is present exclusively on NOR-bearing chromosome arm including pericentromeric region. Interestingly, the sequential Ag-staining and C-banding together with CMA_3_ fluorescence showed that NOR sites stained negative after C-banding procedure. Such an identical association of positive Ag-, CMA_3_ and C- band signals at the NOR sites appears to be ubiquitous pattern for fish genomes. However, our present results for *C.strumicae* showing negative C-bands at NOR sites together with the same findings in *C.vardarensis* (Rábová et al. 2001), *C.elongatoides* (Ráb et al. 2000) and *C.taenia* (Boroň et al. 2006) may indicate the different structural organization of chromosomes at the NOR sites in the genomes of the genus *Cobitis*.

### Cytotaxonomy of *Cobitisstrumicae*

Diploid chromosome number (2n), karyotype structure, i.e. number of chromosomes in the particular categories and especially number and location of NORs, i.e. NOR phenotypes, have proven useful for fish cytotaxonomy. Ráb and Slavík (1996) and Arai (2011) overviewed all available data regarding chromosome studies of *Cobitis* loaches. However, many of listed studies did not provide exact localities, morphological descriptions, data about deposition of voucher specimens and/or depiction of analysed material and what`s more – many reports analysed species under the collective name *C.taenia*. This is the reason why data concerning the name of species given in that list must be used with caution for cytotaxonomic comparisons. As a result, many data should be verified and completed by the new data. Anyhow, the lists of Ráb and Slavík (1996) and Arai (2011) show that only one of the currently recognized species of the subgenus Bicanestrinia was subjected to karyotype analysis: *C.linea* from the Kor River basin, Iran, where authors reported 2n = 50 and a karyotype composed of 4 m, 40 sm and 6 st, NF value 94 (Esmaeili et al. 2015). This karyotype composition differs remarkably from that of *C.strumicae*, but one should bear in mind that both *C.strumicae* and *C.linea* belong to different mitochondrial lineages *sensu*Bohlen et al. (2006) and such variation might indicate the existence of a karyotype differentiation within Bicanestrinia, similarly as within *Cobitis* s. s. (Janko et al. 2007, Ráb et al. 2007).

The species under study, *C.strumicae*, shares the diploid chromosome number 2n = 50 with most of the species karyotyped so far. Its karyotype dominated by uniarmed (acrocentric) chromosomes and lack of morphologically differentiated sex chromosomes is rather common among *Cobitis* loaches.
